# Sinapisms to the Mammæ for Suppression of the Menses

**Published:** 1852-06

**Authors:** O. J. Phelp


From the Ohio Med. Journal.

*Sinapisms to the Mammæ for Suppression of the Menses.* By O. J. Phelp, M. D.

With the view of calling the profession to a more close observation of the sympathies existing between distant organs, I will briefly state a case which recently came under my notice and treatment.

A young woman, 17 or 18 years of age, had begun to suffer seriously from catamenial suppression; and after the use of the ordinary means without success, my last and successful prescription was a repetition of the pediluvium, and sinapisms to the mammæ.

The effect followed almost with telegraphic celerity, the flow following immediately upon the first impression made by the sinapisms, and quite to the astonishment of the mother; and when I intimated to her that I would communicate the case to a medical journal, she said she had been thinking herself that it ought to be done.

Now whether it was the sinapisms alone that produced the effect, or whether it was in part produced by previous treatment, must remain in doubt; but *I* cannot entertain a doubt that the sinapisms were a very strong auxiliary, if not the prime cause, of so decided an effect.

I have now given the facts of the case, upon which might be based a long dissertation on sympathies, &c.; but it would be disrespectful, if not an insult to the profession, to lecture them upon the physiological sympathies existing between the mammæ and uterus. But I am not so certain but there is a great deal yet to be learned of the pathological, and, if such an expression may be used, therapeutical sympathies of those parts.

